# Diminished Returns of Educational Attainment on Numeracy Score of Latino Populations: Insights from UAS Data

**DOI:** 10.31586/ojn.2024.1098

**Published:** 2024-11-05

**Authors:** Shervin Assari, Hossein Zare, Golnoush Akhlaghipour, Mario F Mendez

**Affiliations:** 1 Marginalized-Related Diminished Returns (MDRs) Research Center, Los Angeles, CA, USA; 2 Department of Family Medicine, Charles R Drew University of Medicine and Science, Los Angeles, CA, USA; 3 Department of Urban Public Health, Charles R Drew University of Medicine and Science, Los Angeles, CA, USA; 4 Department of Health Policy and Management, Johns Hopkins Bloomberg School of Public Health, Baltimore, MD, USA; 5 School of Business, University of Maryland Global Campus (UMGC), Adelphi, USA; 6 Paul R. Korey Department of Neurology, Montefiore Medical Center, Bronx, NY, USA; 7 Department of Neurology, University of California Los Angeles (UCLA), Los Angeles, CA, USA; 8 Department of Psychiatry & Biobehavioral Sciences, University of California Los Angeles (UCLA), Los Angeles, CA, USA

**Keywords:** Numeracy Skills, Cognitive Function, Educational Attainment, Minorities’ Diminished Returns, Latino, Health Disparities, Structural Inequality

## Abstract

**Background:**

Educational attainment is a well-established social determinant of various domains of cognitive function across the lifespan. However, the theory of Minorities’ Diminished Returns (MDRs) suggests that the health benefits of educational attainment tend to be weaker for ethnic minorities compared to non-Latino Whites. This phenomenon may reflect the impact of structural inequalities, social stratification, and historical disadvantage.

**Objective:**

This study examines whether the association between educational attainment and numeracy score, one domain of cognitive function, is weaker in Latino individuals compared to non-Latino individuals, as predicted by the MDRs framework.

**Methods:**

Data were drawn from the 2014 wave of the Understanding America Study (UAS), a national internet-based panel. Numeracy score, a domain of the cognitive function was measured using an 8-item measure. Linear regression models were used to analyze the association between educational attainment and numeracy score, with an interaction term for ethnicity × educational attainment to explore differences between Latino and non-Latino participants. Models were adjusted for age, gender, marital status, immigration, and employment, and results were presented as beta coefficients, p-values, and 95% confidence intervals (CIs).

**Results:**

Overall, 5,659 participants entered our analysis. Higher educational attainment was positively associated with higher numeracy score for both Latino and non-Latino participants (p < 0.001). However, the interaction between education and ethnicity was significant (p < 0.05), indicating that Latino individuals experienced smaller numeracy benefits from education compared to non-Latino individuals. These results support the MDRs framework, suggesting that structural barriers may reduce the numeracy returns of education for Latino individuals.

**Conclusion:**

This study provides evidence of diminished returns of educational attainment in terms of numeracy scores among Latino individuals. While education is a key determinant of cognitive abilities such as numeracy, its benefits are not equitably distributed across ethnic groups. Structural inequalities particularly in educational opportunities likely contribute to this disparity. Addressing these underlying factors through targeted policy interventions is necessary to promote cognitive equity for Latino populations.

## 1. Background

Cognitive function, which includes abilities such as numeracy (ability to understand and handle numbers), memory, attention, and problem-solving, plays a crucial role in determining individuals’ quality of life and economic well-being throughout the lifespan [1, 2]. Strong cognitive abilities such as numeracy skills [3–5] are linked to higher levels of economic productivity, better decision-making, and enhanced social engagement over the life course [6, 7]. As societies and jobs increasingly depend on cognitive skills and complex tasks such as handling money, number, and calculations, while routine tasks are automated, cognitive functions such as numeracy skill becomes even more critical. Individuals with higher cognitive performance are better able to maintain their independence and quality of life, whereas those with lower cognitive function are at increased risk of neurodegenerative diseases and other poor health outcomes [8]. As such, cognitive functions such as numeracy are widely regarded as essential for overall well-being and successful human development [5, 9].

Educational attainment is recognized as one of the most significant social determinants of cognitive function [10–13] and numeracy skills [4]. Individuals with more years of schooling and higher levels of educational attainment typically demonstrate better cognitive outcomes, including enhanced memory, attention, executive functioning [10–13], and numeracy skills [4]. Both the quantity and quality of education contribute to the development of cognitive reserve, the brain’s ability to adapt and cope with damage or age-related decline [14–19]. Numerous studies have shown that each additional year of education provides an incremental protective effect against cognitive decline, reinforcing the idea that education acts as a buffer against age-related cognitive challenges [20–27].

While education is generally beneficial across a wide range of human outcomes, the extent of these benefits is not equally experienced across ethnic groups [28]. The theory of Minorities’ Diminished Returns (MDRs) suggests that the positive effects of socioeconomic resources, particularly education, on outcomes like income and employment are less pronounced for ethnic minorities compared to non-Latino Whites [29]. This means that even when marginalized groups attain similar levels of education in quantitative terms, they often experience fewer improvements in health [30], cognitive function [31], and economic outcomes [32] due to disparities in the quality of education and opportunities. These ethnic disparities at the same levels of education reflect broader structural and societal inequalities rooted in social marginalization. MDRs limit the capacity of minority populations to fully convert their educational attainment into optimal life outcomes both in the US [33–35] and globally [36].

Existing studies have demonstrated MDRs in various domains, including health behaviors, mental health, and physical health [29]. For instance, research has shown that while higher education increases income for both White and Black individuals, the financial returns of education are consistently lower for Black individuals [32]. This can be attributed in part to highly educated Black individuals working in less favorable jobs and attending lower-quality schools compared to their White counterparts [37–39]. Similarly, the protective effects of educational attainment on obesity [40], heart disease [41], diet [42], substance use [43], and mental health [44] outcomes (e.g., depression, anxiety, and suicide) are weaker for Latino and Black populations than for their White counterparts.

Although MDRs have been observed across a wide range of outcomes [44–50], limited research has investigated how MDRs might specifically affect cognitive functions such as numeracy skills, particularly in Latino populations [31, 51]. This gap in knowledge is significant, given that Latino individuals represent a large and growing proportion of the U.S. population, and the number of highly educated Latino individuals is increasing. Understanding how the cognitive returns of education differ between Latino and non-Latino groups is crucial for addressing educational and health disparities. While some research has explored MDRs of education on cognitive function across age groups [31], these studies primarily focus on Black versus White individuals. The limited focus on Latino individuals highlights a critical area for further study.

## 2. Methods

### 2.1. The Understanding America Study (UAS)

The Understanding America Study (UAS) [80] is a national internet-based panel survey conducted by the University of Southern California (USC) [52–56]. It aims to provide comprehensive insights into various social, economic, and health-related issues within the U.S. population. Panel members are recruited through probability-based sampling from post-office delivery sequence files. To ensure inclusivity, individuals without internet access are provided with internet-enabled tablets and internet service, enabling broad participation. At the time of data collection, the panel comprised over 9,600 members, including nearly 5,000 individuals aged 50 years or older. The UAS collects a wide range of background variables from all panel members, including information on well-being, retirement planning, personality traits, and cognitive functioning. These core surveys are administered either annually or biennially, alongside repeated cognitive assessments, making participants well-practiced in completing online surveys.

### 2.2. Consent and Ethics

Although all participants were existing members of the UAS panel and had previously consented to participate in UAS studies, the USC Institutional Review Board (IRB) required an expanded consent process for this specific study. The consent survey explicitly stated that individuals diagnosed with progressive cognitive impairments, which might interfere with their ability to provide informed consent, were not eligible to participate. To ensure participants fully understood the study, they were required to answer three multiple-choice questions about their rights as research participants correctly before providing consent. This study received approval from the USC IRB. Respondents were paid $20 to complete the survey.

### 2.3. Analytical Sample Selection

Data for this study were drawn from the UAS in 2014 (first wave), focusing on a subset of panel members. Participants were adults. We did not exclude any participants based on race, ethnicity, or age. Eligibility was valid data on ethnic background (Latino vs non-Latino) and numeracy score.

### 2.4. Cognitive Assessment (Numeracy Skills)

The numeracy scale in the UAS consists of 8 items taken from Weller and colleagues developed in the year 2013 [81]. This measure asks participants to solve problems designed to measure their ability to understand, manipulate, and use numerical information, including probabilities. Items are scored dichotomously as correctly solved or incorrect. In the UAS panel, the numeracy scale scores are derived using a two-parameter logistic Item Response Theory (IRT) model. In this IRT model, the probability of correctly solving a test item is viewed as a function of a test taker’s ability level and the difficulty and discrimination parameters of the test item. The difficulty parameter measures the ability level at which there is a 50% chance of answering the item correctly, whereas the discrimination parameter measures how sensitive this probability is to differences in the ability level. The two-parameter logistic model allows both the difficulty and discrimination parameters to differ across test items.

All items used in the numeracy scale are shown in Appendix 1. These items are designed for both telephone and web-based administration to accommodate the survey format. This scale generated a continuous measure with higher score was indicative of higher numeracy skills [4]. Literature has shown that numeric skill or competency is closely associated with socioeconomic status, and individuals with higher SES have higher numeracy scores. It is also shown that numeric competency prior to school entry enrolled in lower math track classes in high school and were less likely to enroll in college.

### 2.5. Data Analysis

To compare cognitive (numeracy) performance across ethnic groups, we first conducted independent sample t-tests to assess mean differences in numeracy scores. We then ran linear regression models with numeracy score as the dependent variable and educational attainment, ethnicity, age, gender, immigration, employment, and marital status as independent variables. Two models were constructed: Model 1: Baseline model with education, ethnicity, as predictors, without any interaction terms. Model 2: An interaction model that included the interaction term between ethnicity and educational attainment to test whether the effect of education on cognitive function varied by ethnicity. Both models controlled for all confounders. For each regression model, we reported beta coefficients, p-values, and 95% confidence intervals (CIs). This approach allowed us to determine whether there were significant differences in the returns of educational attainment on cognitive function between ethnic groups, with a particular focus on understanding Minorities’ Diminished Returns (MDRs) for Latino populations compared to non-Latino groups.

## 3. Results

Table 1 presents descriptive statistics for the overall sample and by ethnicity (non-Latino and Latino). Significant differences between the groups were observed across several demographic characteristics. The mean age of the overall sample was 49 years (SD = 16), with non-Latino individuals being older on average (M = 50, SD = 15) compared to Latino individuals (M = 39, SD = 14). Regarding education, the overall sample had an average of 11.11 years of education (SD = 2.28). Non-Latino individuals reported higher educational attainment (M = 11.22, SD = 2.22) than Latino individuals (M = 10.21, SD = 2.58). Numeracy scores also differed significantly by ethnicity, with the overall sample averaging a score of 51.12 (SD = 8.43). Non-Latino participants had higher numeracy scores (M = 51.67, SD = 8.37) compared to Latino participants (M = 46.74, SD = 7.56). In terms of gender distribution, the overall sample comprised 45.1% men and 54.9% women. Among non-Latino participants, 46.5% were men and 53.5% were women, whereas the Latino sample had a higher proportion of women (66.6%) compared to men (33.4%). Marriage rates were higher among non-Latino participants, with 62.2% being married, compared to 52.8% of Latino participants. Labor market participation was relatively similar between the groups, with 58.6% of non-Latino participants and 60.6% of Latino participants being employed. Finally, the majority of the overall sample was U.S.-born (94.1%). However, a significantly higher percentage of Latino participants were foreign-born (27.3%) compared to non-Latino participants (3.2%).

Table 2 presents the results of a linear regression model predicting numeracy score, in the absence of any interaction term. Education was a strong positive predictor of numeracy score (b = 1.417, SE = 0.044, 95% CI [1.331, 1.502], p < 0.001), indicating that individuals with more years of education tended to score higher on numeracy. Additionally, being Latino was associated with lower numeracy scores compared to non-Latino individuals (b = −3.426, SE = 0.338, 95% CI [−4.088, −2.763], p < 0.001). Being employed was positively associated with higher numeracy scores (b = 0.661, SE = 0.218, 95% CI [0.235, 1.088], p = 0.002), as was being married (b = 0.961, SE = 0.203, 95% CI [0.562, 1.359], p < 0.001).Age was negatively associated with numeracy score (b = −0.039, SE = 0.007, 95% CI [−0.053, −0.026], p < 0.001), suggesting that older individuals had lower numeracy scores. There was no significant relationship between immigrant status and numeracy (b = −0.410, SE = 0.435, 95% CI [−1.263, 0.443], p = 0.346).

Table 3 presents the results of a linear regression model predicting numeracy score, including an interaction term between Latino ethnicity and educational attainment. Education was a strong positive predictor of numeracy (b = 1.501, SE = 0.047, 95% CI [1.409, 1.593], p < 0.001), suggesting that individuals with more years of education had higher numeracy scores. The interaction between Latino ethnicity and education indicated a negative moderating effect (b = −0.587, SE = 0.122, 95% CI [−0.826, −0.347], p < 0.001), suggesting that the positive impact of education on numeracy was weaker for Latino participants compared to non-Latino participants. Being employed was positively related to numeracy scores (b = 0.688, SE = 0.217, 95% CI [0.262, 1.114], p = 0.002), as was being married (b = 0.936, SE = 0.203, 95% CI [0.538, 1.334], p < 0.001). Age was negatively associated with numeracy score (b = −0.039, SE = 0.007, 95% CI [−0.053, −0.025], p < 0.001), indicating that older individuals had lower numeracy scores. Similarly, being female was associated with lower numeracy scores (b = −3.791, SE = 0.199, 95% CI [−4.181, −3.401], p < 0.001). Immigrant status did not show a significant relationship with numeracy score (b = −0.148, SE = 0.438, 95% CI [−1.007, 0.710], p = 0.735).

## 4. Discussion

This study aimed to examine whether the benefits of educational attainment in terms of numeracy scores differ between Latino and non-Latino individuals, using data from the Understanding America Study (UAS). The hypothesis was grounded in the theory of Minorities’ Diminished Returns (MDRs) [29, 32, 57–61], which suggests that the positive health effects of socioeconomic resources, such as educational attainment, are weaker for marginalized groups, including ethnic minorities, compared to their non-minority counterparts. Specifically, we hypothesized that although higher educational attainment would be associated with higher numeracy scores for both Latino and non-Latino individuals, the strength of this association would be weaker for Latino individuals, reflecting the concept of diminished returns.

Our findings support the hypothesis, revealing that while educational attainment is positively associated with numeracy scores for both Latino and non-Latino individuals, the returns of educational attainment in terms of numeracy skills are indeed smaller for Latino individuals. This result aligns with the MDRs framework and suggests that structural factors may limit the full realization of educational benefits for Latino populations. Additionally, the interaction between education and ethnicity remained significant even after controlling for key demographic factors, such as age, gender, marital status, immigration, and employment, underscoring the persistence of these disparities.

A substantial body of literature has consistently demonstrated the positive effects of education on cognitive function and numeracy scores across the lifespan [20–23, 26, 62–66]. Higher levels of educational attainment are associated with improved numeracy scores [4] as well as other domains of cognitive reserve [14, 20, 23, 63, 67–69], better memory, and stronger problem-solving abilities, which can protect against cognitive decline in later life. Education is thought to contribute to cognitive health by promoting intellectual engagement, problem-solving skills, and social and economic advantages that may mitigate cognitive decline as individuals age. Numerous studies have shown that each additional year of education is associated with better cognitive outcomes [20–23], reflecting the critical role education plays in sustaining cognitive functioning in older adulthood.

Research on Minorities’ Diminished Returns (MDRs) has highlighted that the protective effects of educational attainment and other socioeconomic resources are often weaker for ethnic minorities [29, 58, 59, 61, 70–73]. Studies have documented diminished returns of education on outcomes such as income, mental health, and physical health. In terms of cognitive function, a few studies have shown that ethnic minority individuals tend to receive fewer cognitive benefits from education compared to their White counterparts. This suggests that structural barriers, including systemic racism and social marginalization, reduce the extent to which ethnic minority individuals can convert educational attainment into cognitive health. However, while MDRs have been extensively studied among Black populations, there is a paucity of research on MDRs among Latino individuals, particularly regarding cognitive function.

The literature on MDRs in Latino populations is limited, and to our knowledge, no studies have specifically investigated diminished cognitive returns of education among Latino individuals. While previous research has demonstrated that Latino individuals face similar structural barriers as other ethnic minorities, including discrimination, fewer economic opportunities, and lower access to quality education, the direct impact of these factors on the cognitive benefits of education has not been thoroughly explored [74–79]. Our study is among the first to document evidence of diminished cognitive returns of educational attainment for Latino individuals, contributing to the growing body of literature on MDRs and highlighting the need for more research in this area.

Several potential mechanisms may explain why Latino individuals experience diminished cognitive returns from education. Structural inequality is likely a key factor, as systemic barriers restrict access to quality educational resources, health care, and economic opportunities, even for those with higher educational attainment. Additionally, Latino individuals may experience higher levels of chronic stress due to discrimination, economic insecurity, and social marginalization, which can negatively affect cognitive health over time. Further, poor nutrition, higher rates of food insecurity, and lower access to health-promoting resources may compound the negative effects of these stressors. Together, these factors create an environment in which the returns of education, particularly for cognitive health, are limited for Latino individuals.

### 4.1. Implications

The findings of this study have significant implications for public health and educational policy. Efforts to improve numeracy skills and cognitive health among Latino individuals must go beyond increasing access to education and should address the broader structural inequalities that limit the effectiveness of education in promoting cognitive function. Additionally, tailored public health strategies that address nutrition, healthcare access, and job training opportunities could help improve cognitive outcomes in Latino populations.

### 4.2. Limitations

This study has several limitations that should be considered when interpreting the findings. First, the sample was limited to English-speaking UAS participants, which may not fully capture the diversity of the Latino population in the United States, particularly those who speak primarily Spanish or have lower levels of acculturation. Second, while we controlled for key demographic factors, unmeasured confounders, such as childhood socioeconomic conditions and access to early education, may have influenced the results. In addition, this study did not investigate the heterogeneity of the Latino populations based on culture, country of origin, immigration status, etc. Finally, the cross-sectional nature of the data limits our ability to draw causal conclusions about the relationship between educational attainment and cognitive function. Longitudinal studies are needed to confirm the patterns observed here and to explore how these associations may change over time.

## 5. Conclusion

In conclusion, this study provides evidence of diminished returns from educational attainment in terms of numeracy skills among Latino individuals, supporting the broader theory of Minorities’ Diminished Returns (MDRs). While educational attainment is a key determinant of numeracy and cognitive health, the benefits of education are not equitably distributed across ethnic groups. Structural inequalities, chronic stress, and adverse socioeconomic conditions likely play significant roles in limiting the cognitive benefits of education for Latino individuals. Addressing these disparities will require multi-level interventions targeting both individual and structural factors to improve cognitive outcomes and promote health equity.

## Figures and Tables

**Table 1.** Descriptive Data Overall and by Ethnicity

	All (n = 5659)		Non-Latino (n =5030)		Latino (n = 629)	
	Mean	Std. Deviation	Mean	Std. Deviation	Mean	Std. Deviation
Age (Yrs) *	49	16	50	15	39	14
Education Years*	11.11	2.28	11.22	2.22	10.21	2.58
Numeracy Score (Yr 12)*	51.1	8.4	51.7	8.4	46.7	7.6
	n	%	N	%	n	%
Ethnicity						
Non-Latino	5030	88.90	5030	100	-	-
Latino	629	11.1	-	-	629	100
Gender*						
Men	2551	45.1	2341	46.5	210	33.4
Women	3108	54.9	2689	53.5	419	66.6
Married*						
No	2198	38.8	1901	37.8	297	47.2
Yes	3459	61.1	3127	62.2	332	52.8
In Labor market*						
No	2329	41.2	2081	41.4	248	39.4
Yes	3329	58.8	2948	58.6	381	60.6
US-Born*						
No	335	5.9	163	3.2	172	27.3
Yes	5324	94.1	4867	96.8	457	72.7

* p < 0.05 for comparison of Latino and non-Latino people

**Table 2.** Summary of the linear regression model with the interaction term

Model	Unstandardized Coefficients		Standardized Coefficients	Sig.	95.0% Confidence Interval for B	
	B	Sth. Error	Beta		Lower Bound	Upper Bound
Age (Years)	−.039	.007	−.073	< .001	−.053	−.026
Gender (Woman)	−3.794	.199	−.224	< .001	−4.185	−3.403
Immigrant	−.410	.435	−.011	.346	−1.263	.443
Working	.661	.218	.039	.002	.235	1.088
Married	.961	.203	.056	< .001	.562	1.359
Education (Years)	1.417	.044	.384	< .001	1.331	1.502
Ethnicity (Latino)	−3.426	.338	−.128	< .001	−4.088	−2.763

Dependent Variable: Numeracy Score

**Table 3.** Summary of the linear regression model with the interaction term

Model	Unstandardized Coefficients		Standardized Coefficients	Sig.	95.0% Confidence Interval for B	
	B	Std. Error	Beta		Lower Bound	Upper Bound
Age (Years)	−.039	.007	−.072	< .001	−.053	−.025
Gender (Woman)	−3.791	.199	−.224	< .001	−4.181	−3.401
Immigrant	−.148	.438	−.004	.735	−1.007	.710
Working	.688	.217	.040	.002	.262	1.114
Married	.936	.203	.054	< .001	.538	1.334
Education (Years)	1.501	.047	.407	< .001	1.409	1.593
Ethnicity (Latino)	2.713	1.323	.101	.040	.119	5.307
Latino × Education	−.587	.122	−.231	< .001	−.826	−.347

Dependent Variable: Numeracy Score

## Appendix 1. Items used for measurement of ǹumeracy

**Table:**

Imagine that we roll a fair, six-sided die 1,000 times. Out of 1,000 rolls, how many times do you think the die would come up as an even number? RANGE 0..1000 In the BIG BUCKS LOTTERY, the chances of winning a $10.00 prize are 1%. What is your best guess about how many people would win a $10.00 prize if 1,000 people each buy a single ticket from BIG BUCKS? RANGE 0..1000 lip003 In the ACME PUBLISHING SWEEPSTAKES, the chance of winning a car is 1 in 1,000. What percent of tickets of ACME PUBLISHING SWEEPSTAKES win a car? RANGE 0.0..100.0 If the chance of getting a disease is 10%, how many people would be expected to get the disease out of 1000? RANGE 0..1000 If the chance of getting a disease is 20 out of 100, this would be the same as having how much of a percent chance of getting the disease? RANGE 0..9223372036854775807 Suppose you have a close friend who has a lump in her breast and must have a mammogram. Of 100 women like her, 10 of them actually have a malignant tumor and 90 of them do not. Of the 10 women who actually have a tumor, the mammogram indicates correctly that 9 of them have a tumor and indicates incorrectly that 1 of them does not have a tumor. Of the 90 women who do not have a tumor, the mammogram indicates correctly that 80 of them do not have a tumor and indicates incorrectly that 10 of them do have a tumor. The table below summarizes all of this information. Imagine that your friend tests positive (as if she had a tumor), what is the likelihood that she actually has a tumor? A bat and a ball cost $1.10 in total. The bat costs $1.00 more than the ball. How much does the ball cost? NUMBER (DECIMALS ALLOWED) In a lake, there is a patch of lily pads. Every day, the patch doubles in size. If it takes 48 days for the patch to cover the entire lake, how long would it take for the patch to cover half of the lake? NUMBER (DECIMALS ALLOWED)
